# Human skill knowledge guided global trajectory policy reinforcement learning method

**DOI:** 10.3389/fnbot.2024.1368243

**Published:** 2024-03-15

**Authors:** Yajing Zang, Pengfei Wang, Fusheng Zha, Wei Guo, Chuanfeng Li, Lining Sun

**Affiliations:** ^1^State Key Laboratory of Robotics and System, Harbin Institute of Technology, Harbin, China; ^2^School of Electronics and Information Engineering, Harbin Institute of Technology, Harbin, China

**Keywords:** path planning, imitation learning, reinforcement learning, behavioral cloning, probabilistic movement primitives

## Abstract

Traditional trajectory learning methods based on Imitation Learning (IL) only learn the existing trajectory knowledge from human demonstration. In this way, it can not adapt the trajectory knowledge to the task environment by interacting with the environment and fine-tuning the policy. To address this problem, a global trajectory learning method which combinines IL with Reinforcement Learning (RL) to adapt the knowledge policy to the environment is proposed. In this paper, IL is proposed to acquire basic trajectory skills, and then learns the agent will explore and exploit more policy which is applicable to the current environment by RL. The basic trajectory skills include the knowledge policy and the time stage information in the whole task space to help learn the time series of the trajectory, and are used to guide the subsequent RL process. Notably, neural networks are not used to model the action policy and the Q value of RL during the RL process. Instead, they are sampled and updated in the whole task space and then transferred to the networks after the RL process through Behavior Cloning (BC) to get continuous and smooth global trajectory policy. The feasibility and the effectiveness of the method was validated in a custom Gym environment of a flower drawing task. And then, we executed the learned policy in the real-world robot drawing experiment.

## 1 Introduction

Trajectory learning is crucial in robot task learning, encompassing most applications in the field of robotics, especially in industrial robotics where learning specific movement skill trajectories is fundamental.

Traditional task trajectory learning methods primarily rely on imitation learning. Imitation learning based on mathematical modeling can achieve simplified representation and reproduction of specific shape trajectories by extracting their mathematical characteristics. This is commonly used for teaching robots specific actions like shaking, picking, drawing, etc. Later, with the rise of neural networks, methods based on neural networks could approximate action skill sampling strategies directly through BC, or approach human global strategies using Generative Adversarial Imitation Learning (GAIL) methods that differentiate between human and neural network strategies. Inverse Reinforcement Learning (IRL) methods also approximate human strategies through exploration. These methods have achieved good results in imitating, reproducing, and generalizing human trajectories.

However, these methods are all based on human operation as a benchmark. They learn skills that more accurately represent human operation to complete task strategies but do not interact with the environment to correct and optimize actions. The reality is, not all human operations are optimal. Therefore, these methods cannot learn strategies beyond human skills, meaning they cannot achieve strategy exploration and discovery based on the environment, making it difficult to perform effective strategy optimization.

In methods that enable global policy optimization, the exploration and reward mechanisms in RL are powerful tools for policy exploration and discovery (Zhao et al., 2023; Bing et al., 2023a,b, 2022a), commonly used in autonomous learning tasks for robotic operations. However, a significant drawback of RL is the difficulty in exploring the continuous and dense policy space, which under a time-series context, resembles an endless ocean. Finding a trajectory that meets specific requirements is particularly challenging. Therefore, it's widely believed that using RL to learn skill trajectories is impractical due to the enormous number of explorations required. Despite this, the exploration and discovery functions of RL are very appealing for policy optimization. Consequently, guided RL methods have emerged, aiming to constrain the exploration learning process of RL through skill knowledge, to achieve rapid learning of basic human strategies and subsequent optimization.

Up to now, knowledge-guided RL methods have been significantly applied in robot task learning. However, the methods vary across different tasks, including using expert strategies to replace some random exploration strategies, ensuring full use of knowledge, narrowing the exploration range based on knowledge to reduce irrational exploration actions, or constructing special reward functions to guide learning toward specific actions. These methods optimize and modify RL through existing task knowledge, integrating prior knowledge into the RL process for task-specific optimization, thereby enhancing learning efficiency or quality.

Based on this concept, we propose a knowledge-guided trajectory policy learning optimization method. This method extracts mathematical characteristics of demonstration trajectories in traditional imitation learning, including shape, temporal, and reasonable exploration range features, and uses these features to guide the basic policy learning and optimization in RL. Unlike traditional RL methods, we record action strategies, advantageous action libraries, and action values through sampling during the exploration learning process, rather than using neural networks. After policy iteration learning based on sampled states, the learned strategies are recorded in neural networks through BC, achieving continuous representation of strategies and providing a basis for sampling continuous, smooth task trajectories.

Our contributions are as follows:

1. Sample the state point in the task space with DTW method for trajectory alignment.
2. Establish the basic knowledge policies and the time stages by IL, which are used to guide the subsequent RL.
3. A RL method guided by human skill knowledge, based on sampled action policies and action values, is proposed to achieving global policy learning and optimizing in the task space.
4. Using BC to obtain continuous action policies from global policy samples for generating proper robot flower drawing task trajectories.

The paper is organized as follows: Section 2 discusses related work. Section 3 introduces the extraction methods of human skill knowledge, including the basic trajectory skills, and the action exploration range. Section 4 describes the task space sampling RL method guided by skill knowledge. Section 5 covers the reinforcement learning experiments and result analysis, along with real-world robot experiments. Finally, Section 6 concludes the paper.

## 2 Related work

### 2.1 Trajectory learning method based on imitation learning

Trajectory learning based on imitation methods is mainly divided into those based on mathematical analysis and those based on neural networks. The traditional mathematical analysis methods primarily work by establishing mathematical models to learn and reproduce trajectories. For example, the attractor models of trajectory and force are based on the Dynamic Movement Primitives (DMP) method, the probabilistic models of trajectory parameters are based on Probabilistic Movement Primitives (ProMPs), and the time-related trajectory point probability models are based on Time-Parameterized Gaussian Mixture Models (TP-GMM). The application of the DMP includes using DMP to establish trajectory and force profile models in Liao et al. (2023), to learn the stiffness profiles of the robot in robot compliance control (Bian et al., 2020), and to improve the human-robot handover tasks in Wang et al. (2021a). The employment of ProMPs involves applying ProMPs to human-robot interaction scenarios in Koert et al. (2019), where they are used to achieve obstacle avoidance by changing shapes and time scaling. A general probabilistic adaptive method is also proposed in Frank et al. (2022), which provides a unified ProMPs framework for multiple complex robotic tasks like obstacle avoidance, via-points, and mutual avoidance. What's more, a method combining the advantages of DMP and ProMPs is also provided in Li et al. (2023b), in which the ProMPs is embedded into neural networks to achieve efficient end-to-end learning of advanced trajectory statistics. The TP-GMM method, in Rozo et al. (2015); El Zaatari et al. (2021); Duque et al. (2019) for instance, establishes a Gaussian mixture model by extracting probability models under the task and base coordinate systems, then reproduces trajectories through the Gaussian Mixture Regression (GMR). In this way, the method could learn the characteristics of the trajectory in specific task frame. Overall, the IL methods based on mathematical analysis can effectively extract data parameters of the trajectory, such as the shape and the via-points of the trajectories. However, the limitation of them is that they can only reproduce the existing trajectory features based on the mathematical models but cannot learn extra trajectory policy related to the task environment in the whole task space.

IL methods based on neural networks like BC, utilizes the policy samples obtained from human demonstration to train neural networks for policy learning. For example, it is used in Li et al. (2023a) to learn autonomous driving technologies from the human driving skills. It is also applied to learn robot assembly task in Zang et al. (2023). Even though BC method has the potential to model the global task strategy, it may also be hard for it to learn extra policy to adapt to the environment. While the Generative Adversarial Imitation Learning (GAIL) method aims at learning the human task strategy by training the generators and discriminators to interact with each other. In the previous study, GAIL are used to learn human driving strategies in Bhattacharyya et al. (2023), to learn human navigation trajectories in Fahad et al. (2020), and robot assembly tasks in Jiang et al. (2023); Gubbi et al. (2020). Inverse reinforcement learning (IRL) methods design specific reward function for the target task to allow RL agents to approximate human strategy under the attraction of the task reward. It has been used for learning the navigation strategies in Herman et al. (2015); Xia and El Kamel (2016), ping pong playing strategies in Muelling et al. (2014), and robot force-related tasks in Hussein et al. (2019).

### 2.2 Knowledge guided reinforcement learning method for robot task

Nowardays, RL has been widely used to help robot learn task strategies (Bing et al., 2022b,c, 2023c). And knowledge-guided RL methods related to robot task learning are mainly divided into exploration strategy planning, network parameter initialization, and the setting of special reward functions.

Among them, exploration strategy planning is the most common method. Since most of the current knowledge exists in the form of policy actions, improving the exploration process of reinforcement learning based on expert strategies is a direct and effective method of guidance for reinforcement learning (Subramanian et al., 2016; Ma et al., 2021). An expert system is introduced to multi-agent reinforcement learning methods to guide strategy exploration, thereby improving the learning efficiency of reinforcement learning in Wang et al. (2022b). Nair A and others from the University of California (Sharma et al., 2019) avoid unreasonable random exploration by restarting from demonstrated actions. However, this exploration method also does not achieve optimization of the exploration strategy. Nicolas and others from the National Institute of Information and Communications Technology in Japan (Bougie et al., 2018) optimize the exploration strategy by training neural networks to choose advantageous decisions. Although they have achieved optimization of the exploration strategy, the optimization of exploration strategies based on neural networks often struggles with generalization.

Parameter initialization refers to the pre-training of the agent with skill parameters or the direct assignment of values to reinforcement learning-related variables before the reinforcement learning process begins, allowing the agent to initially master task skills (Kim et al., 2020). Taylor ME and others at Edgewood Technical Institute assigned initial values to demonstration action values based on demonstrated operations (Taylor et al., 2011). However, due to the sparsity of the demonstration value parameters, they cannot provide comprehensive guidance for reinforcement learning. In Bendikas et al. (2023), tasks are recursively decomposed into a series of subtasks, and then the agent is initialized with the existing critic network parameters to guide the current actor, thus achieving the guiding effect of the network Q function. In Wang et al. (2021b) and Wang et al. (2022a), for the control of complex assembly tasks, imitation learning is used to initially learn the outline of the trajectory, and then its parameters are used for subsequent force control learning, resulting in effective assembly force control strategies.

The setting of the reward function is a very important part of the reinforcement learning process. Thus, setting specific reward functions according to the characteristics of the task, enabling the reinforcement learning agent to learn knowledge-biased trajectories, is one of the methods of knowledge-guided reinforcement learning. For example, Guo et al. (2023) uses an innovative composite auxiliary reward structure and a Soft Actor-Critic with Self-Paced Prioritization (SAC-SP) mechanism to realize optimal feedback control in real-time. In Gu et al. (2023), an artificial potential field is used to set the reward function, allowing the reinforcement learning agent to learn robot trajectories that avoid obstacles. The same artificial potential field reward method is also used in Xue et al. (2023) to guide the reinforcement learning agent to learn avoidance behaviors. Ao et al. (2023) designs a practical reward function for unmanned aerial vehicle (UAV) applications, taking into account the throughput, safety distance, and power consumption of the UA virtual machine.

## 3 Human skills extraction method

In the work of Zang et al. (2023), we modeled demonstration trajectories using the ProMPs imitation learning method based on mathematical analysis. We then estimated the current state's time stage using probabilistic methods and set the midpoint of the next time stage as the target point to obtain a target position strategy based on position control. Finally, we learned the pin insertion assembly strategy based on position control using the BC method. In this paper, we will use the same method to extract basic strategies, but we have made some improvements in the implementation details, and the specific implementation methods are as follows.

### 3.1 Basic knowledge extraction

In order to provide initial strategy guidance and time stage guidance for the reinforcement learning process, we also use the method in Zang et al. (2023) to segment the demonstration information by time stages and extract knowledge strategies, considering them as the fundamental trajectory knowledge.

In this paper, we focus on knowledge-guided reinforcement learning for two-dimensional trajectories. Therefore, we set the collection of demonstration trajectories as *Y*, with each trajectory being two-dimensional but of varying lengths, denoted as *Y*_1:2_. Due to the varying time progression of human demonstration information, we process *Y* using DTW to obtain the processed two-dimensional trajectory sequence $Y_{1:2}^{DTW}\left(N\right)$, where *N* represents the length of the aligned trajectory timeline. Additionally, the distance function of DTW is set as the Euclidean distance between two sampling points, meaning for any two sequence points ^1^*P*^*DTW*^ and ^2^*P*^*DTW*^, their distance is expressed by the Equation 1.


(1) $$\begin{array}{l} Dist=|{|}^{1}Y_{1:2}^{DTW}{-}^{2}Y_{1:2}^{DTW}|| \end{array}$$

For each dimension *k*∈{ 1, 2} of the geometric representation $Y_{1:2}^{DTW}$, we establish probability motion primitives, expressed as Equations 2, 3.


(2) $$\begin{array}{l} y_{k}^{DTW}\left(t\right)=\Phi_{k}w_{k}+\epsilon_{y_{k}^{DTW}} \end{array}$$

(3) $$\begin{array}{l} P\left(\tau_{k}|w_{k}\right)=\underset{t}{\Pi }N\left(y_{k}^{DTW}\left(t\right)|\Phi_{k}w_{k},\;\Sigma_{y_{k}^{DTW}}\right) \end{array}$$

Where $\Phi_{k}\in {\mathbb{R}}^{n}$ are the basis functions of the four-dimensional geometric representation variable of ProMPs, and *n* represents the number of these basis functions. *w*_*k*_ is the weight vector corresponding to the basis function. $\epsilon_{y_{k}^{DTW}}\sim N\left(0,\;\Sigma_{y_{k}^{DTW}}\right)$. Then, we use the same method as in Zang et al. (2023) to calculate the Gaussian model parameters θ_*k*_ = {μ_*w*_*k*__, Σ_*w*_*k*__} of the weight parameters.

When dividing the trajectory into time stages, we still adopt the method from Zang et al. (2023). First, we set the maximum distance for each time stage of the trajectory *l*_*thre*_, as well as the maximum time step *t*_*thre*_, ensuring that all points within the same time stage satisfy the Equation 4. Then, for the mean trajectory $Y_{mean}^{DTW}\left(t\right)$ generated based on ProMPs, we traverse from the first point to the last point. As soon as any point fails to satisfy either condition of Equation 4, we start a new time stage from that event point, making it *t*_*start*_, while the previous point *t*_*end*_ becomes the end of the previous time stage.


(4) $$\{\begin{array}{c} \forall t\in T_{s},||Y_{1:4}^{DTW}(t_{s})-Y_{1:4}^{DTW}(t_{start})||\leq l_{thre}\\ t_{end}-t_{start}\leq t_{thre} \end{array}$$

Then, for all the trajectories, we use all the sampling points of each time stage to calculate the Gaussian model θ_*T*_*s*__ = {μ_*T*_*s*__, Σ_*T*_*s*__} of the trajectory points for that time stage. This model is used to subsequently determine the time stage information for any sampling point.

For any given sampling point *P*^*sample*^, we calculate the probability density *p*_*t*_*s*__ of all the time stage Gaussian models at that sampling point. The time stage corresponding to the highest probability density is considered to be the time stage of that sampling point. However, when generating trajectories with ProMPs, having only the time stages is not sufficient. Therefore, we always assume that the time point in which the sampling point is located is represented by the Equation 5.


(5) $$\begin{array}{l} t=\text{int}\left(\frac{t_{end}\text{+}t_{start}}{2}\right) \end{array}$$

Based on the estimated time points and the positions of the sampling points, we set the sampling points for ProMPs and then generate the target trajectory. In the generated target trajectory, we set the target position as the mean of some sampling points in the next time stage. To ensure sufficient task progress in the guided trajectory, the end time of this part of the sampling points is set to the *t*_*end*_ of the next time stage, while the starting time point is set to any time point on either side of the midpoint of the next time stage. We designate the final target point as *P*^*sample*^, thereby obtaining the current knowledge strategy *a*_*know*_ defined as Equation 6.


(6) $$\begin{array}{l} a_{know}=P^{target}-P^{sample} \end{array}$$

Due to the randomness in time sampling, it also ensures that our strategy does not converge at the beginning, thus providing sufficient learning space for the subsequent reinforcement learning process.

### 3.2 The exploration range extraction

**Figure 1:**
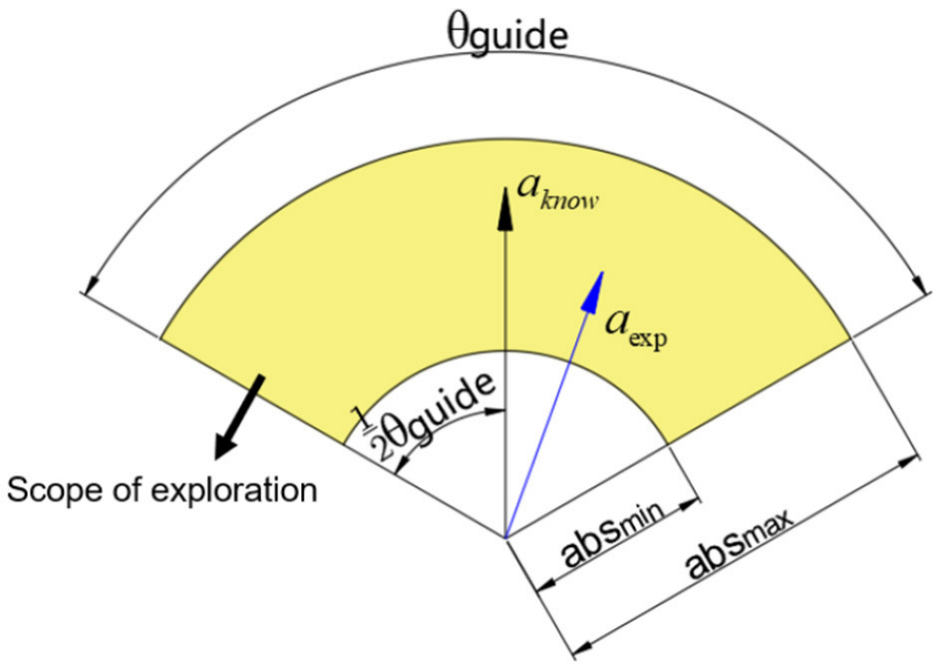
The picture of the guided exploration range.

After determining the knowledge strategy for the sampling points, we will guide the exploration behavior according to the direction and magnitude of the knowledge strategy. We assume that the knowledge strategy at the sampling point *P*^*sample*^ is *a*_*know*_. When exploring in a two-dimensional environment, to ensure the general direction of the exploration trajectory, we assume that the size of the exploration angle range is θ_*guide*_, and the size range of the exploration action length is [*abs*_min_, *abs*_max_]. We represent the exploration range as Figure 1.

Assuming there are two random values ε_θ_∈[−0.5, 0.5], ε_*l*_∈[0, 1], and assuming the direction vector of the knowledge strategy *a* is *v*_*a*_, then we can determine that the exploration action *a*_exp_ needs to revolve around the policy *a*_*know*_ with the rotation matrix given by the Equation 7.


(7) $$\begin{array}{l} T_{guide}=\left[\begin{array}{cc} \cos\varepsilon_{\theta }\theta_{guide} & -\sin\varepsilon_{\theta }\theta_{guide}\\ \sin\varepsilon_{\theta }\theta_{guide} & \cos\varepsilon_{\theta }\theta_{guide} \end{array}\right] \end{array}$$

The length of the *a*_exp_ can be denoted as Equation 8.


(8) $$\begin{array}{l} l_{guide}=\varepsilon_{l}\left(abs_{\max}-abs_{\min}\right)+abs_{\min} \end{array}$$

Then, the *a*_exp_ can be denoted as Equation 9.


(9) $$\begin{array}{l} a_{\exp}=l_{guide}\cdot T_{guide}v_{a} \end{array}$$

## 4 Knowledge-guided reinforcement learning method with sampled task states

In the previous chapter, we acquired task knowledge including information about time stages, exploration knowledge strategies, and exploration ranges. In this chapter, we will dedicate ourselves to utilizing this knowledge to guide the reinforcement learning process and complete the learning of trajectory drawing tasks.

### 4.1 Sampling states in task space

Unlike traditional reinforcement learning methods that learn directly in a continuous task space, in this paper, we will perform spatial sampling in a two-dimensional task space and learn at limited, discrete sampling points. This is because, although traditional continuous spaces theoretically allow for random strategy learning across the entire task space, such learning is based on a large number of explorations and is not conducive to practical operations. At the same time, with the constant updating of policies and value functions in reinforcement learning, it is not easy to achieve the convergence of both policy and value functions to a better strategy. Especially for tasks like trajectory learning that do not require a very complex distribution of strategies, using sampled states for reinforcement learning is a very feasible and time-saving approach.

First, we divide the two-dimensional task space into many equal areas *R*_*task*_ as the basic areas for our strategy learning, and for each area, we determine its corresponding time stage $T_{s}^{R}$. To focus on learning effective sampling points, we will determine the sampling density based on the distribution of demonstration trajectories. For the number of real demonstration trajectory samples in an area *R*_*task*_, we set certain thresholds, thus classifying them into areas with different sampling densities. For example, if the number of real demonstration trajectory samples *N*_*real*_ in an area *R*_*task*_ is 0, we can set the number of sampling points to 1, i.e., we only set one sampling point *s*^*sample*^ in the center of the area, and $N_{R}^{s^{sample}}=1$. If the number of real demonstration trajectory samples is 0 < *N*_*real*_ ≤ 50, we can set an average of 4 sampling points *s*^*sample*^ in these areas, with $N_{R}^{s^{sample}}=4$, and so on. Thus, we have sampled different areas of the task space with different densities based on the demonstration trajectories.

### 4.2 Knowledge guided reinforcement learning

In the guided learning based on sampled states, we set the strategy as a determined strategy for all sampling points *s*^*sample*^, denoted as *a*^*s*^^*sample*^. However, unlike traditional reinforcement learning which establishes an experience replay pool, we do not use a randomly sampled experience replay pool. Instead, we use a similarly sampled experience strategy library *L*^*s*^^*sample*^. This experience strategy library stores several advantageous strategies for each state sampling point, ensuring that the agent does not forget some advantageous actions due to the learning process not yet converging. We set an upper limit on the number of experience strategies stored for all sampling points as $N_{\max}^{L}$, ensuring that the storage space we use and the computational load during optimization are both controllable.

In our study, the value function is also sampled based on the distribution of the sampling points. Thus, for each state sampling point, we establish a deterministic policy *a*^*s*^^*sample*^, an action value *Q*^*s*^^*sample*^, and an experience strategy library *L*^*s*^^*sample*^.

During the exploration process, we conduct action exploration for all the sampling points we have set. Firstly, we constrain the exploration actions according to the method described in Section 3.1, selecting suitable exploration actions. By conducting explorations from the same starting point and choosing the exploration action with higher rewards, we update the policy *a*^*s*^^*sample*^ and simultaneously refresh the experience strategy library, achieving constrained random exploration for the task. Additionally, we search for superior strategies within a small range around the existing experience strategies, replacing the original strategies to fine-tune the experience strategy library, known as minor local exploration. Through constrained random exploration and minor local exploration, we can offer a sampling-based guided reinforcement learning exploration strategy.

Regarding the calculation method for exploration rewards, we have also made improvements. Firstly, to ensure that the policy trajectory does not involve repetitive cycles, we stipulate that rewards can only be obtained after exploring for a certain number of steps and reaching a time phase beyond the starting point; otherwise, a penalty is incurred. Moreover, the discount factors for the reward calculation include both the current time phase's discount factor and subsequent time stages' discount factors, thereby estimating the agent's potential to obtain more rewards in the current time phase. The final formula for calculating exploration rewards is as Equation 10.


(10) $$G_{a_{\exp}}^{s^{sample}}\text{=}\{\begin{array}{c} \sum \gamma_{1}{\;}^{t-1}R_{t}^{T_{s}}+\gamma_{2}Q_{s^{\prime }}\\ -R_{punish} \end{array}\begin{array}{c} if\;\exists T_{s^{\prime }}>T_{s}\\ if\;\forall T_{s^{\prime }}\leq T_{s} \end{array}$$

Here, $R_{t}^{T_{s}}$ represents the reward obtained at the t-th time step after starting from the initial sampling point. *R*_*punish*_ is a fixed value. $Q_{s^{\prime }}$ denotes the action value of the sampling point when the agent reaches the subsequent time phase's sampling point *s*′.

During the process of policy updating, we calculate the reward value for each exploration action, and use the obtained advantageous actions and advantage experience library to update the old policy and experience library. In the updating process, we adopt an updating method that incorporates the idea of temporal difference learning. The formula is as Equations 11, 12.


(11) $$\begin{array}{ll} a^{s^{sample}} & =\left(1-\alpha_{a}\right)a^{s^{sample}}+\alpha_{a}a_{dom}^{s^{sample}} \end{array}$$

(12) $$\begin{array}{ll} L^{s^{sample}} & =\left(1-\alpha_{L}\right)L^{s^{sample}}+\alpha_{L}L_{dom}^{s^{sample}} \end{array}$$

In this context, $a_{dom}^{s^{sample}}$ represents the best action determined after random and local exploration, yielding the highest reward. On the other hand, $L_{dom}^{s^{sample}}$ refers to a set of advantageous actions identified post-exploration, which are characterized by higher rewards. α_*a*_ and α_*L*_ are the update coefficients for the policy and experience strategy library, respectively.

**Figure 2:**
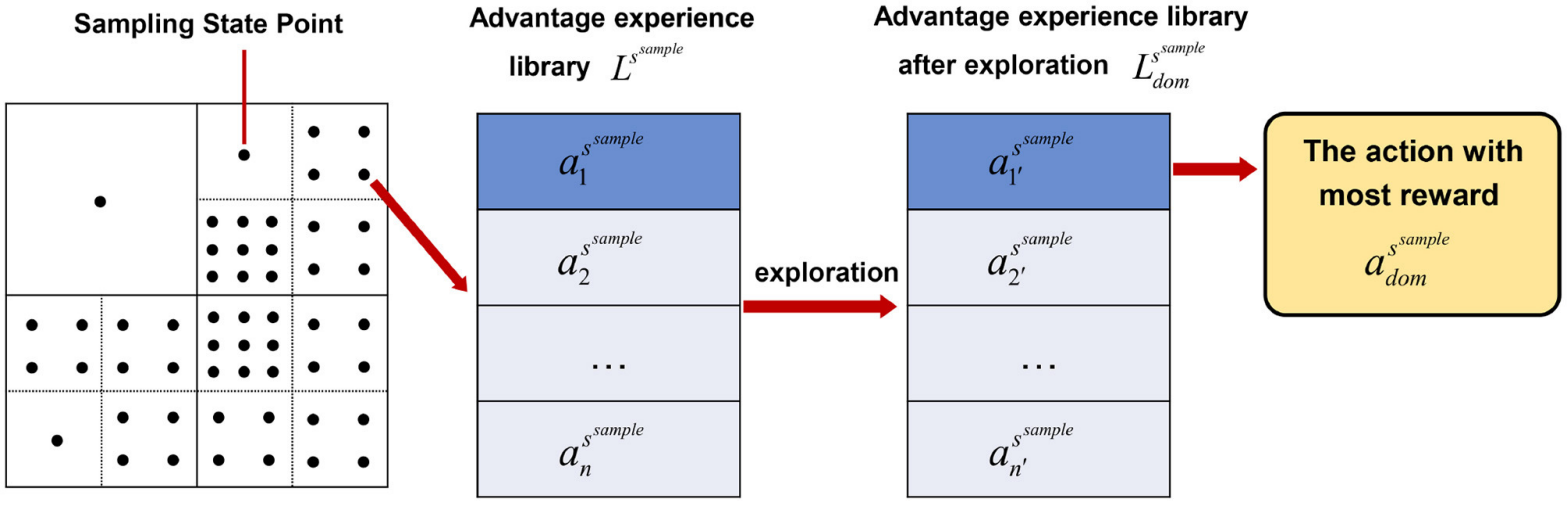
The relationship of the proposed variables *a*^*s*^^*sample*^, *L*^*s*^^*sample*^ and $L_{dom}^{s^{sample}}$.

Finally, to present the relationship of the proposed variables *a*^*s*^^*sample*^, *L*^*s*^^*sample*^ and $L_{dom}^{s^{sample}}$ more clearly, Figure 2 is given in follows.

### 4.3 Behavior cloning for sampled policy

After several iterations of guided reinforcement learning, we obtain a converged set of exploration action samples. However, these cannot be directly used as the final policy, as such a policy is discrete and fails to produce a continuous, smooth trajectory. Therefore, we model the acquired policy through behavior cloning. By employing the behavior cloning method, the derived policy can be approximated using a neural network, resulting in a continuous and smooth policy. Moreover, since our policy is sampled across the entire task space, we can also train it using multiple neural networks of different styles. This approach allows for better approximation effects or smaller network structures.

## 5 Experiments and evaluation

**Figure 3:**
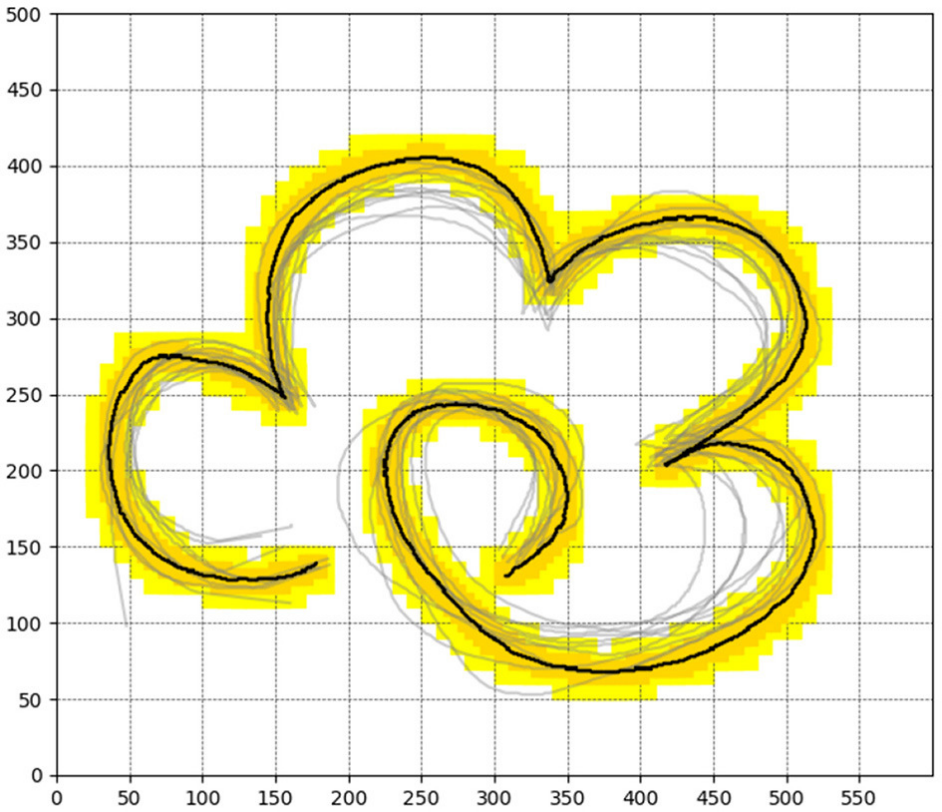
The reward distribution of the flower drawing environment as well as the human demonstration trajectories.

To validate the effectiveness of our proposed sampling-based guided reinforcement learning method, we conducted reinforcement learning iterative experiments and real robot experiments. Firstly, we set up a flower trajectory drawing experiment and established a Gym environment for the task. The schematic diagram of the environment is shown in Figure 3.

In this setup, the task space is 600 units long and 500 units wide. The black part represents the standard trajectory, while the dark yellow and light yellow parts are the trajectory's extension areas. The rewards are allocated as follows: the highest reward is set for the black part at 100, the dark yellow part has a reward of 2, and the light yellow part has the lowest reward, set at 0.05. The reward for all other parts is set to 0. It's important to note that the agent do not have prior knowledge of the reward distribution in the environment before learning, the guidance information can be obtained according to the gray demonstration trajectory. Additionally, to ensure the continuity of the trajectory, our rewards are also only for one-time, which means that the reward for a specific location will not be granted a second time.

### 5.1 Acquisition of trajectory skills

**Figure 4:**
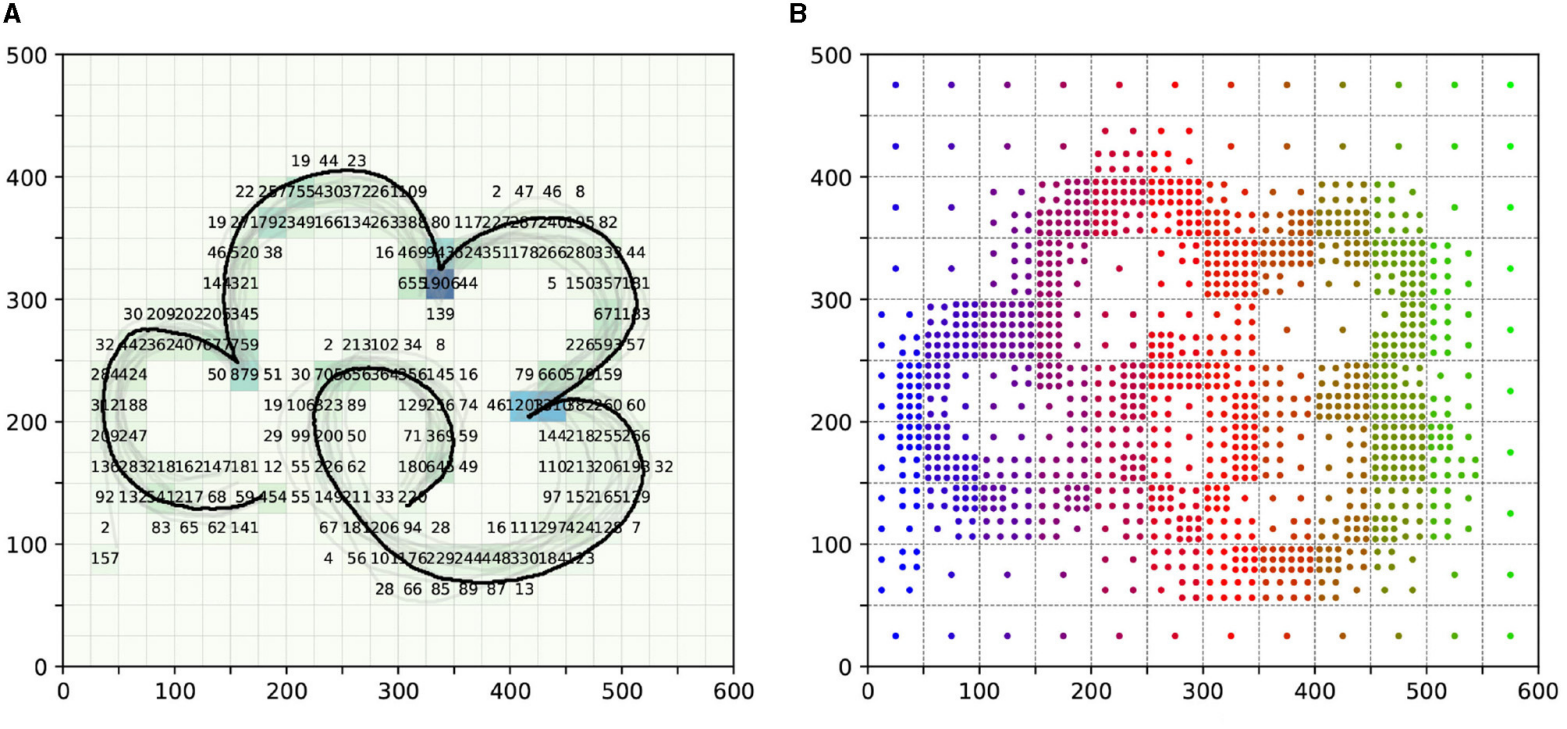
The sampling process of task space. **(A)** is the number of sampling points of demonstration information in each area. **(B)** is the final sampling condition of the task space. Notably, the color of the sampling points in **(B)** is only used to distinguish the different areas of the division.

Firstly, to sample the states within the task space, we divided the task space into several 25 × 25 units. We then counted the number of sampling points of demonstration information in each area, as shown in Figure 4A. After determining the number of sampling points, we proceeded with the sampling of the task space, as illustrated in Figure 4B.

**Figure 5:**
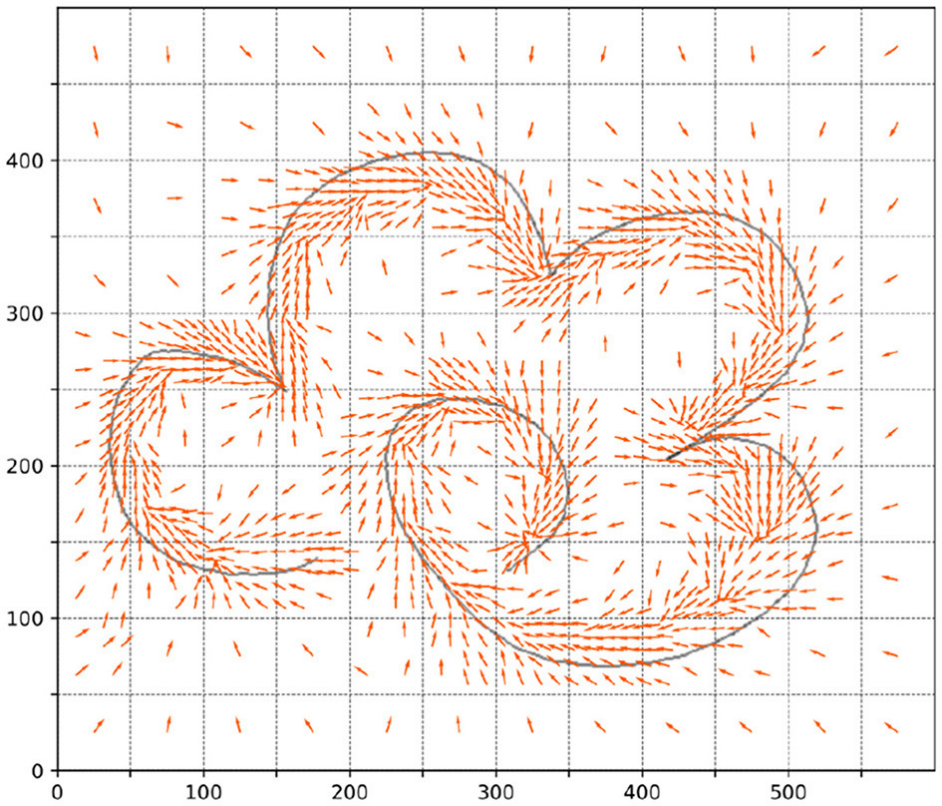
The knowledge policy of each sample point in each area of the task space.

According to the method in Section 3.1, we extract the knowledge policy of each sample point in each area of the task space, as shown in Figure 5.

**Figure 6:**
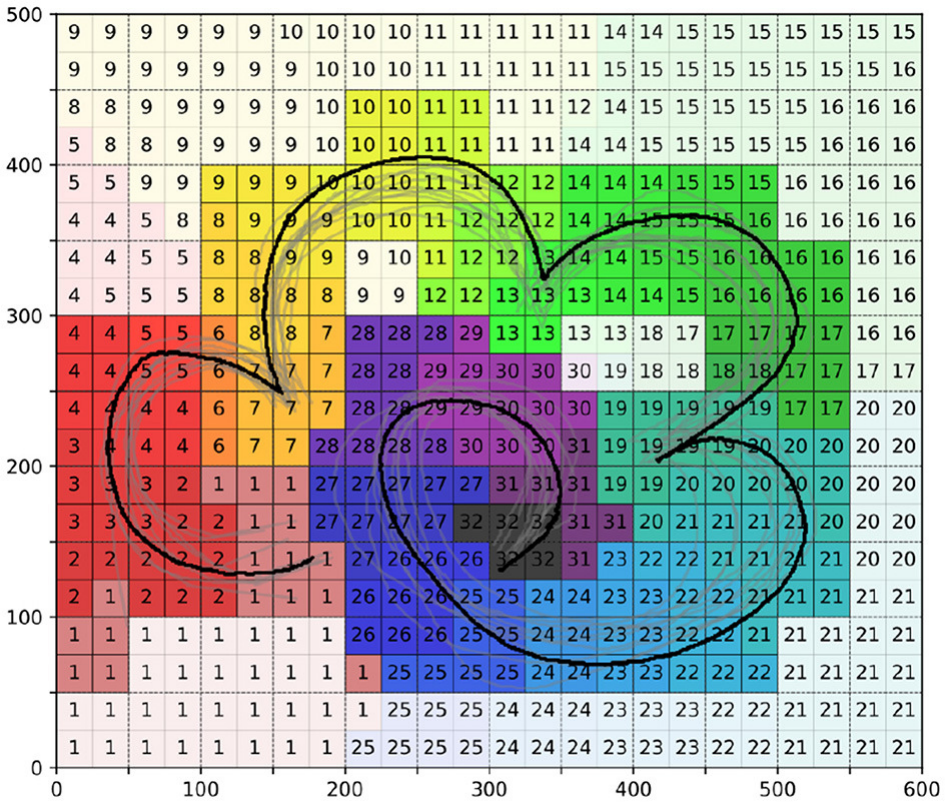
The time stages of different areas in the task space.

Additionally, we obtained information about the time stages of different areas in the task space, as depicted in Figure 6. In this figure, the darker areas represent time phase regions near the demonstration information, while the lighter areas are time phase regions further from the demonstration information. During the learning process, we will first focus on strategy learning in the darker regions, followed by the lighter regions.

### 5.2 Knowledge guided reinforcement learning experiment

We utilized the empirical knowledge obtained in Section 5.1 for the subsequent guided reinforcement learning. According to the time phase segmentation in Section 5.1, we conducted action exploration for each sampling point. The exploration related parameter θ_*guide*_ is set to $\frac{2}{3}\pi$. As for parameter *l*_*guide*_, since *abs*_*min*_ and *abs*_*max*_ are 2 and 4 respectively, *l*_*guide*_ is in between. In this way, each exploration action can be determined according to Section 3.2. For each exploration, the maximum number of steps was set to 50. For each sampling point, we carried out 10 constrained random explorations and 5 minor local explorations. Completing the exploration process for each sampling point constitutes one complete iteration. After 100 complete iterations, we found that the policy could achieve convergence.

To validate the superiority of our proposed method, we conducted the following comparative experiments:

1. Guided trajectory RL with demonstration density sampling states + BC (Our method).
2. Naive trajectory RL with demonstration density sampling states + BC.
3. Partially Guided DDPG trajectory RL with continuous states.
4. Naive DDPG trajectory RL with continuous states.
5. Knowledge-based strategy only + BC.

's important to note that our guided reinforcement learning method cannot be fully applied to the DDPG method. Therefore, in experiment 3, we only used the network parameters from experiment 5 to initialize the DDPG Actor network and used the exploration range constraints obtained from Section 3.2 to restrict exploration behavior. To allow the Critic network's parameters to adapt to the initial Actor parameters, we did not train the Actor network in the first 100 steps, and these steps were not counted in the learning process. That is, the learning process for experiment 3 began after 100 steps. Additionally, since experiment 5 did not involve iterations, we will directly present its experimental results.

Furthermore, since network parameter initialization was performed in Experiment 3, we set the number of iteration steps to 5,000 for this experiment, while in Experiment 4, it was set to 10,000 steps. In recording, we also noted data at intervals of 50 steps for Experiment 3 and 100 steps for Experiment 4, ultimately obtaining 100 sets of data.

**Figure 7:**
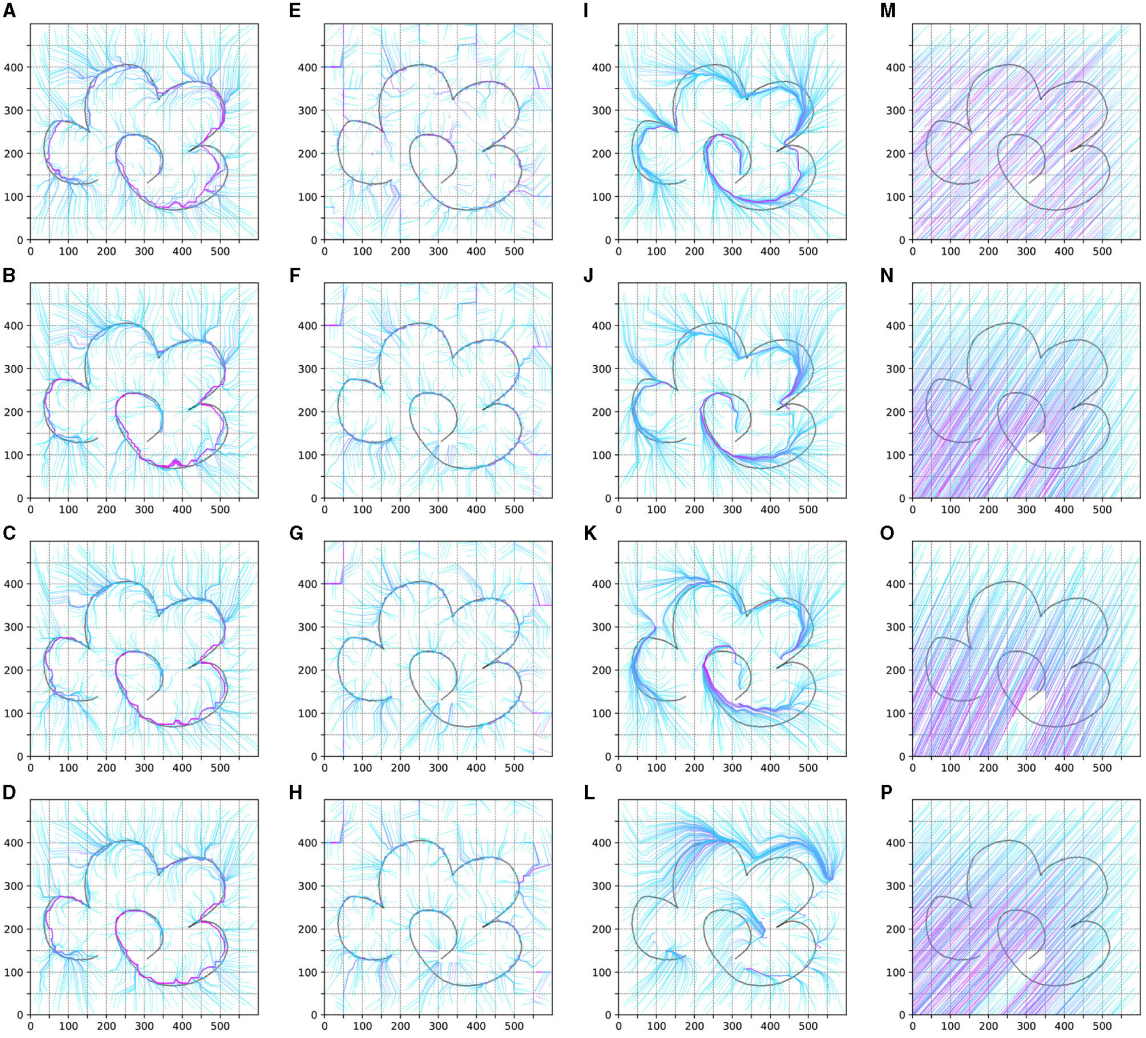
The result of the test trajectory policy of the obtained agents in experiment 1–4. **(A–D)** is the result of experiment 1. **(E–H)** is the result of experiment 2. **(I–L)** is the result of experiment 1. **(M–P)** is the result of experiment 4.

We test the trajectory policy of the obtained agents in experiment 1–4 as shown in Figure 7. It illustrates the policy changes over the learning iteration goes on. For Experiments 1 and 2, we recorded the policy experimental results after the 3rd, 20th, 50th, and 100th complete iterations. For Experiments 3 and 4, we showcased the data from the 3rd, 20th, 50th, and 100th sets. For each set of data, we uniformly sampled 600 trajectories in the entire task space, each trajectory lasting 200 time steps. The figure demonstrates the learning situation of these trajectories.

**Figure 8:**
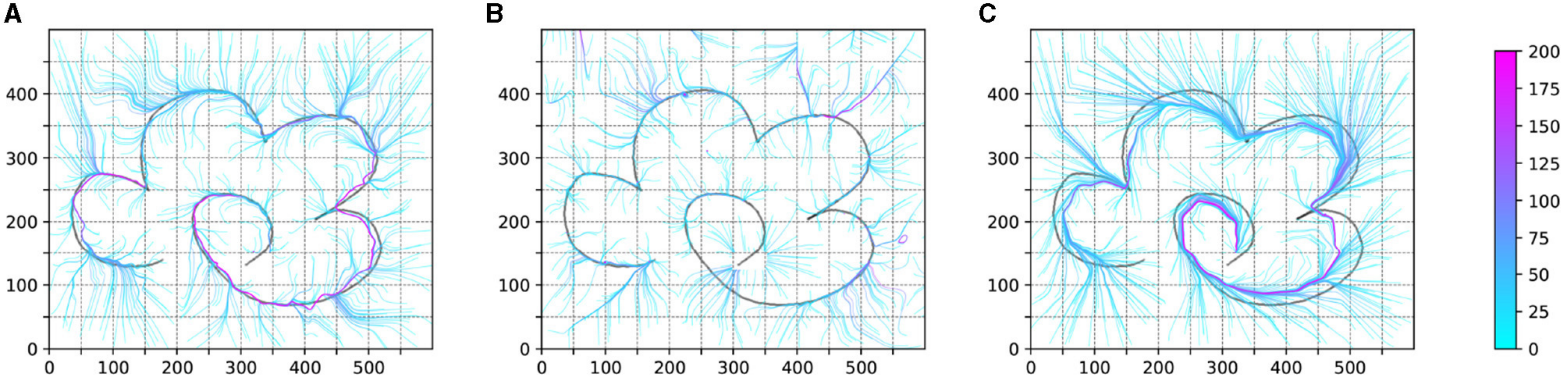
The result of the test trajectory policy of the BC process. **(A, B)** is the result of BC agent obtained in experiment 1 and experiment 2. **(C)** is the result of BC agent obtained in experiment 5.

In Figure 8, we conducted BC for the policies obtained from Experiments 1 and 2, presenting the trajectory test results of the policies represented by neural networks. Additionally, this figure includes the trajectory test results from Experiment 5.

**Figure 9:**
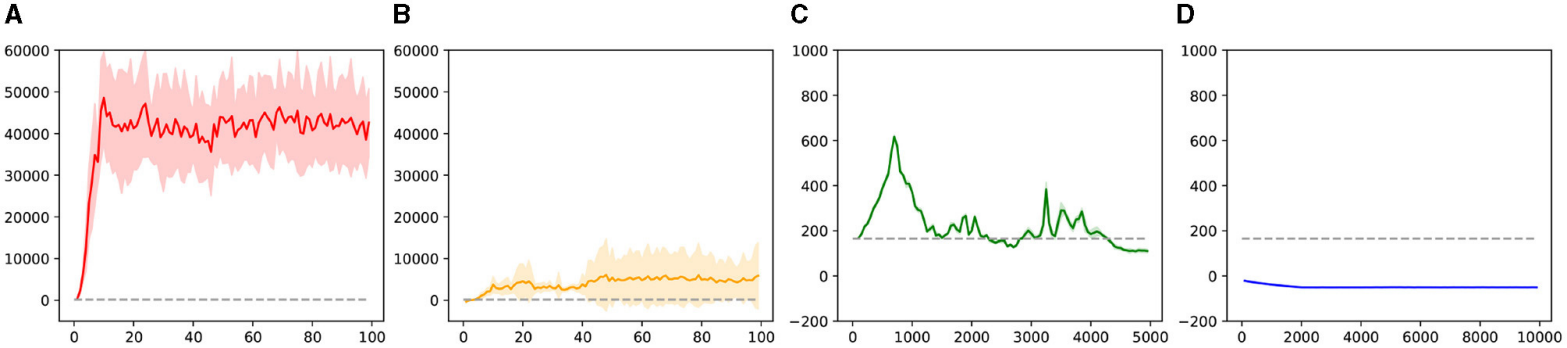
The test results of the environment reward in experiment 1–4. **(A)** is the result of experiment 1. **(B)** is the result of experiment 2. **(C)** is the result of experiment 1. **(D)** is the result of experiment 4.

We also recorded the environmental reward test results for each recorded iteration in Experiments 1–4, as shown in Figure 9. Additionally, we tested the reward results for Experiment 5, which are represented by dashed lines in each subplot.

**Figure 10:**
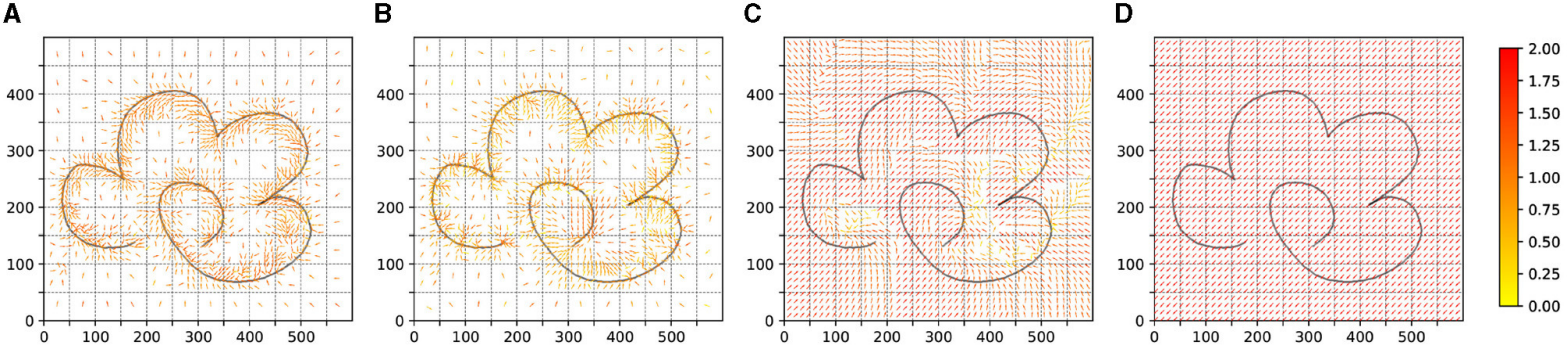
The policy sampling of the test results of experiments 1–4. **(A)** is the result of experiment 1. **(B)** is the result of experiment 2. **(C)** is the result of experiment 1. **(D)** is the result of experiment 4.

We also provide policy sampling diagrams for the results of Experiments 1–4, as illustrated in Figure 10. The policy sampling diagram for Experiment 5, which represents the knowledge strategy sampling, is shown in Figure 4.

**Figure 11:**
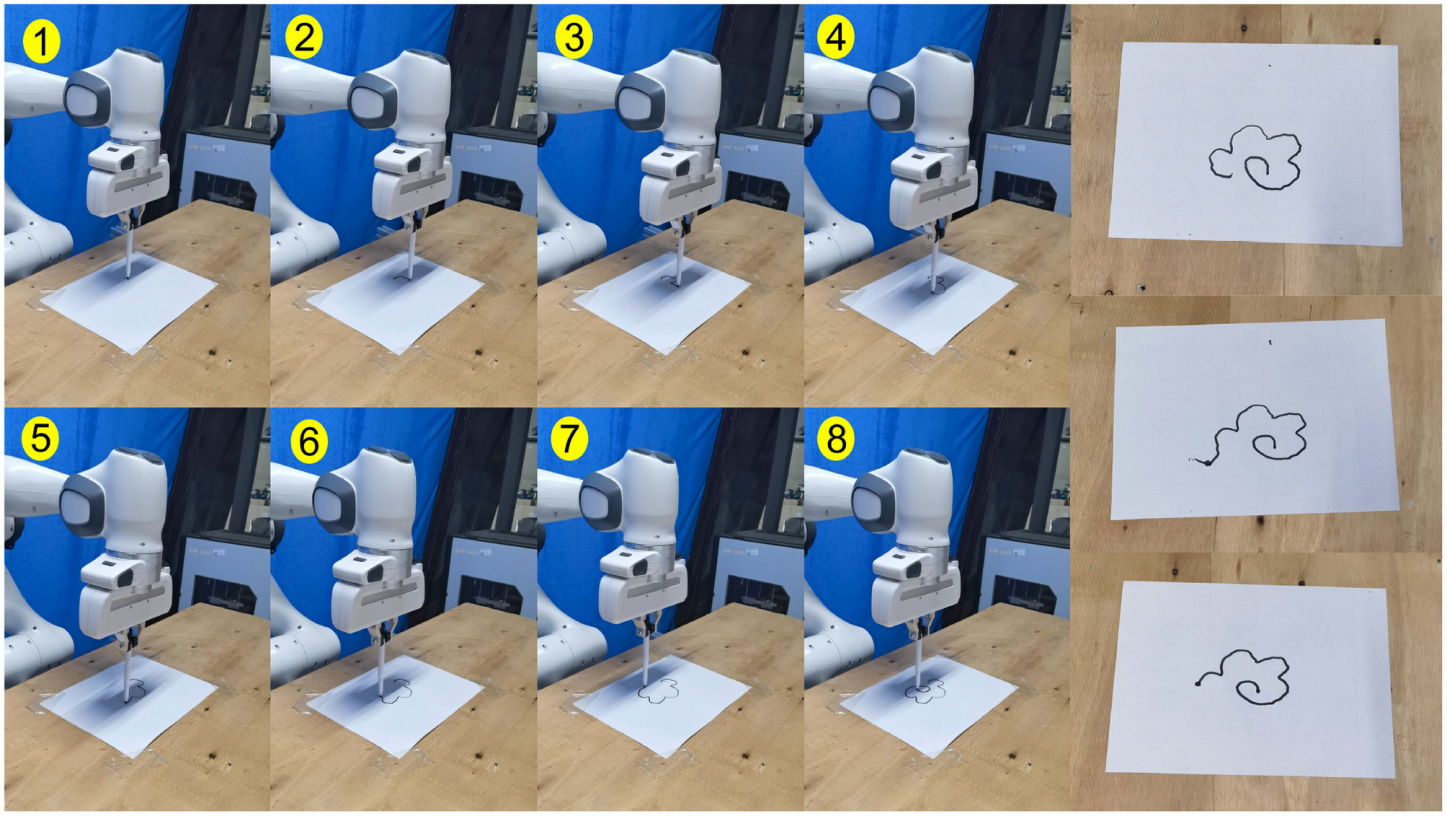
The picture of the real world experiment snapshots and the flower drawing results.

Additionally, based on the continuous policy generated by the BC network in Experiment 1, we generated real robot trajectories and validated their feasibility on a Franka Emika robot. In the experiment, the robot's inverse kinematics were used to calculate the joint angles required for generating two-dimensional end-effector trajectories. Then, a joint position control method was employed to enable the robot to draw the flower trajectory. Snapshots of one experiment and several experimental result images are shown in Figure 11. Furthermore, a video of the experiment will be uploaded to https://youtube.com/shorts/FGWAfngazxk?feature=share.

### 5.3 Results and evaluation

As shown in (Figures 7A–D), under the influence of the knowledge strategy and guided exploration, the reinforcement learning agent can quickly learn useful policies. Through iteration, these policies are optimized, allowing the agent to learn a global strategy capable of generating complete trajectories. This process also leads to better adaptation to the environment and the acquisition of more rewards.

For the unguided sampling-based reinforcement learning method in Experiment 2, while the agent can learn strategies to obtain rewards, the lack of guided policies means it lacks long-term vision. It can only learn strategies concentrated in reward areas, failing to generate complete trajectories and obtaining only limited, unstable rewards.

In the guided DDPG method, thanks to parameter initialization and exploration guidance, the agent initially performs well, learning better strategies in the early stages of the trajectory. However, as this method is neural network-based and cannot sample the entire task space's strategies simultaneously, the agent gradually forgets the initial effective strategies. This leads to slow learning initially and rapid forgetting later, preventing the formation of an effective global strategy.

Regarding the naive DDPG reinforcement learning method, despite having twice the number of iterations compared to the guided methods, it still fails to learn an effective strategy. This difficulty can be attributed to our experimental environment not being fully Markovian, and the temporal task itself requiring extensive exploration to learn basic strategies. Hence, in Experiment 4, the agent struggles to learn strategies in the task space without guidance.

Overall, our method has significant advantages over traditional reinforcement learning approaches in learning basic strategies, optimizing strategies according to environmental rewards, and preserving existing knowledge strategies to prevent neural network knowledge forgetting. Comparing the results of Experiments 2 and 3, we see that sampling-based reinforcement learning indeed helps avoid past pitfalls. Comparing Experiments 3 and 4, we conclude that guided reinforcement learning aids the agent in rapidly mastering basic strategies, leading to quicker learning of effective, environment-adaptive strategies. Additionally, we believe that the guided DDPG reinforcement learning method has the potential to learn more and better strategies, though knowledge forgetting and the tendency of reinforcement learning to fall into local optima make this process slow and challenging.

In the real-world robot task, it is simple to generate some similar trajectories to the demonstration task through the imitation learning method. However, it is difficult to truly learn global task strategies which are more adapted to the environment only through mathematical imitation. This is the reason why we present this approach. Moreover, the experiment also proves that, it is an effective path to achieve task strategy optimization by direct and efficient guided strategy exploration and updating methods to obtain optimization strategies more suitable for the current environment on the basis of basic skills. Finally, the 2D trajectory rendering task also enables the advantages of the method to visualize the experimental results more clearly and clearly, which provides convenience for the verification of the effect of our method.

## 6 Conclusion

In this paper, we propose a guided reinforcement learning method based on the sample density of the demo. Among them, the main contribution is that we do not rely on neural networks to directly model reinforcement learning strategies and action value functions, but through sampling methods to ensure that the learning of global strategies to the task space, at the same time to avoid forgetting knowledge. In addition, task-based knowledge strategies, including constrained random exploration and micro-local exploration, can enable agents to effectively improve exploration strategies, enable intelligence to learn more quickly and more useful task strategies in the current environment.

## Data availability statement

The raw data supporting the conclusions of this article will be made available by the authors, without undue reservation.

## Author contributions

YZ: Conceptualization, Data curation, Formal analysis, Investigation, Methodology, Software, Validation, Visualization, Writing – original draft, Writing – review & editing. PW: Conceptualization, Formal analysis, Funding acquisition, Investigation, Methodology, Project administration, Resources, Supervision, Writing – review & editing. FZ: Conceptualization, Formal analysis, Funding acquisition, Investigation, Methodology, Project administration, Resources, Supervision, Writing – review & editing. WG: Conceptualization, Formal analysis, Funding acquisition, Investigation, Methodology, Project administration, Resources, Supervision, Writing – review & editing. CL: Conceptualization, Data curation, Formal analysis, Software, Writing – original draft. LS: Funding acquisition, Investigation, Resources, Supervision, Writing – review & editing.
